# Do depressive symptoms among pregnant women assisted in Primary Health Care services increase the risk of prematurity and low birth weight?

**DOI:** 10.1590/1518-8345.4932.3480

**Published:** 2021-09-03

**Authors:** Anelise de Toledo Bonatti, Ana Paula dos Santos Costa Roberto, Thais de Oliveira, Milena Temer Jamas, Maria Antonieta de Barros Leite Carvalhaes, Cristina Maria Garcia de Lima Parada

**Affiliations:** 1 Universidade Estadual Paulista “Júlio de Mesquita Filho”, Faculdade de Medicina de Botucatu, Botucatu, SP, Brazil.; 2 Scholarship holder at the Conselho Nacional de Desenvolvimento Científico e Tecnológico (CNPq), Brazil.; 3 Secretaria Municipal de Saúde de Botucatu, Botucatu, SP, Brazil.

**Keywords:** Depression, Pregnancy, Low Birth Weight Infant, Premature Infant, Prenatal Care, Primary Care Nursing, Depressão, Gestação, Baixo Peso ao Nascer, Recém-Nascido Prematuro, Pré-Natal, Enfermagem de Atenção Primária, Depresión; Embarazo, Recién Nacido de Bajo Peso, Recien Nacido Prematuro, Atención Prenatal, Enfermería de Atención Primaria

## Abstract

**Objective:**

to investigate associations between depressive symptoms during pregnancy, low birth weight, and prematurity among women with low-risk pregnancies assisted in public Primary Health Care services.

**Method:**

prospective cohort with 193 pregnant women, using the Edinburgh Postnatal Depression Scale, telephone interviews, and medical records available in the health services. Associations of interest were obtained using the Cox regression model.

**Results:**

the participants were aged 24.9 years old (median) and had 11 years of schooling (median); 82.4% lived with their partners, and gestational age at the birth was 39 weeks (median). Twenty-five percent of the participants scored ≥13 on the Edinburgh scale. Depressive symptoms did not appear associated with low birth weight (RR=2.06; CI95%=0.56-7.61) or prematurity (RR=0.86; CI95%=0.24-3.09) in the adjusted analysis. However, premature labor increased the risk of low birth weight (RR=4.81; CI95%=1.01-23.0) and prematurity (RR=7.70; CI95%=2.50-23.7). Additionally, each week added to gestational age decreased the risk of low birth weight (RR=0.76; CI95%=0.61-0.95).

**Conclusion:**

the presence of depressive symptoms among women with low-risk pregnancies was not associated with low birth weight or prematurity.

## Abstract (pt)

**Objetivo:**

investigar a associação entre sintomas depressivos na gestação, baixo peso ao nascer e prematuridade entre gestantes de baixo risco obstétrico, atendidas em serviços públicos de Atenção Primária à Saúde.

**Método:**

coorte prospectiva com 193 gestantes, mediante aplicação da Escala de Depressão Pós-natal de Edimburgo, entrevista telefônica e consulta aos prontuários dos serviços de saúde. As associações de interesse foram obtidas por regressão múltipla de Cox.

**Resultados:**

as participantes tinham idade mediana de 24,9 anos e escolaridade mediana de 11 anos; 82,4% viviam com companheiro e a idade gestacional mediana no parto foi 39 semanas. Auferiram escore ≥13 na Escala de Edimburgo 25,4% delas. Na análise ajustada, sintomas depressivos não se associaram ao baixo peso ao nascer (RR=2,06; IC95%=0,56-7,61) e à prematuridade (RR=0,86; IC95%=0,24-3,09). Secundariamente, identificouse que trabalho de parto prematuro aumentou o risco de baixo peso ao nascer (RR=4,81; IC95%=1,01-23,0) e de prematuridade (RR=7,70; IC95%=2,50-23,7). Além disso, cada semana a mais na idade gestacional diminuiu o risco de baixo peso ao nascer (RR=0,76; IC95%=0,61-0,95).

**Conclusão:**

a presença de sintomas depressivos entre gestantes de baixo risco obstétrico não se associou ao risco de baixo peso ao nascer e prematuridade.

## Abstract (es)

**Objetivo:**

investigar la asociación entre síntomas depresivos en la gestación con bajo peso al nacer y prematuridad entre embarazadas, de bajo riesgo obstétrico, atendidas en servicios públicos de Atención Primaria a la Salud.

**Método:**

cohorte prospectiva en 193 embarazadas, utilizando la Escala de Depresión Posparto de Edimburgo, por medio de entrevista telefónica y consulta en las fichas médicas de los servicios de salud. Las asociaciones de interés fueron obtenidas con la regresión múltiple de Cox.

**Resultados:**

las participantes tuvieron edad mediana de 24,9 años y escolaridad mediana de 11 años; 82,4% vivían con compañero y la edad gestacional mediana en el parto fue 39 semanas. 25,4% de las mujeres obtuvieron un puntaje ≥13, en la Escala de Edimburgo. En el análisis ajustado, los síntomas depresivos no se asociaron al bajo peso al nacer (RR=2,06; IC95%=0,56-7,61) y a la prematuridad (RR=0,86; IC95%=0,24-3,09). Secundariamente, se identificó que el trabajo de parto prematuro aumentó el riesgo de bajo peso al nacer (RR=4,81; IC95%=1,01-23,0) y de prematuridad (RR=7,70; IC95%=2,50-23,7). Además de eso, se encontró que cada semana a más en la edad gestacional disminuye el riesgo de bajo peso al nacer (RR=0,76; IC95%=0,61-0,95).

**Conclusión:**

la presencia de síntomas depresivos entre embarazadas de bajo riesgo obstétrico no se asoció al riesgo de bajo peso al nacer y a la prematuridad.

## Introduction

Depression is considered a public health problem due to its severity, recurrence, and negative impact on health^(1)^. According to the World Health Organization (WHO), more than 254 million people of all ages are affected by depression worldwide. Hence, depression is among the primary causes of incapacity and significantly contributes to the global load of illness, especially among women^(2)^. Depression is a mental disorder characterized by depressive mood, sleep disorders, lack of interest in daily tasks, difficulty concentrating, guilt, lack of or excessive appetite, and occasionally thoughts and suicidal ideation^(1,3-4)^.

Depression is highly prevalent during pregnancy, affecting one in every five women worldwide^(1)^. The various physical, hormonal, emotional, and psychosocial transformations that occur during pregnancy directly influences these women’s mental health, explaining its prevalence^(4-5)^.

The consequences of depression during pregnancy in the short and long terms have been investigated, emphasizing the harm caused to the fetus’s health, including low birth weight and prematurity. Despite the biological plausibility of this association, studies present conflicting results^(6-9)^.

A systematic review reports an association between prenatal depression and premature birth in less than ¼ of the 50 studies included in the study, and approximately half (53%) of the studies addressing low birth weight found an association with prenatal depression^(6)^. A recent literature review included seven studies in which increased risk of prematurity was found among women with depression, while another five studies did not find such an association^(9)^.

A prospective cohort conducted in 2018 in Kenya, not included in the previous reviews, found an association between depression and premature labor: women with depression during pregnancy were approximately four times more likely to have premature babies, in comparison to women without depressive symptoms^(7)^. The cutoff point adopted in the study for the Edinburgh Postnatal Depression Scale (EPDS) was equal to or higher than 10 points. In the same year, a Vietnamese cohort study using the same scale and cutoff point found a risk three times greater for prematurity and a twice greater risk for low birth weight among women with depression^(8)^.

In Brazil, the relationship between depression during pregnancy, low birth weight, and prematurity is seldom addressed. A cohort study addressing women with low-risk pregnancies was conducted in the capital of São Paulo and did not find any association between depression and low birth weight or prematurity^(10)^. Another cohort study, conducted in a city located in the south of Brazil, addressed women exclusively assisted by the Unified Health System (SUS) during prenatal care and labor and verified that mothers experiencing depression during pregnancy were four times more likely to have an infant with low birth weight^(11)^.

The previous discussion shows no consensus in the international or Brazilian literature regarding the relationship between gestational depression, low birth weight, and prematurity, which justifies this study. This study’s primary objective was to investigate the potential association between depressive symptoms during pregnancy, low birth weight, and prematurity among women with low-risk pregnancies assisted by Primary Health Care services. The secondary objective was to identify the factors associated with both outcomes.

## Method

### Study design

This prospective cohort study was conducted in the mid-south region of the state of São Paulo, Brazil^(12)^.

### Study setting

The study was conducted in Botucatu, SP, Brazil, a city with an estimated population of 146,497 inhabitants in 2019^(13)^. According to data provided by the cities in the state and the *Fundação Sistema Estadual de Análise de Dados* ^(14)^ [State Data Analysis Foundation System], the city’s São Paulo Social Responsibility Index, wealth and longevity dimensions are below those reported by the state of São Paulo. The services sector accounts for 45.3% of the city’s formal jobs.

Botucatu has PHC units that work as the entrance door to the health system: 2 PHC units linked to a University and 18 PHC units linked to the city hall: 6 Basic Health Unit (UBS), and 12 Family Health Units (UFS) with 15 teams. The UBS are a reference in pediatrics, gynecology and obstetrics, and general practice within the covered area, providing care to 63.0% of the population. USF have a team composed of family physicians, nurses, dentists, nursing technicians, dental assistants, and community health agents located in the city’s periphery, providing care to 37.0% of the city’s population^(15)^.

### Study participants

The pregnant women were recruited in all the PHC units in the city between May and December 2018. The following inclusion criteria were used: pregnant women at any gestational age, aged 18+ years old, and literate. Multiple pregnancies were excluded due to the risk of low birth weight and prematurity.

This study addressed a convenience sample composed of 193 pregnant women, who were monitored until child delivery and had all data of interest available.

### Instruments used in data collection

The EPDS, a scale initially developed to investigate postpartum depression^(5)^, was used to collect data concerning depressive symptoms during pregnancy. It was translated into and validated in many countries, including Brazil^(16)^, and was indicated and validated to be used during pregnancy. A systematic review, addressing instruments designed to assess depression among pregnant women and identify the most appropriate instruments to apply in prenatal care services, reports that the EPDS was the most accurate scale (area under the curve ROC=0.965, sensitivity=0.80, and specificity=0.81)^(17)^. Its advantages include the fact that it is a self-reported, simple, and easy-to-apply instrument, simple to be interpreted, and any professional can apply^(4,16)^.

The EPDS is a questionnaire composed of ten topics with four options of answers, ranging from zero to three, according to the absence or presence and intensity of symptoms^(18-20)^. The questions address the presence of depressive or dysphoric mood, guilt, sleep disorders, loss of pleasure, decreased performance, and death and suicide ideation^(4-5)^. The final score ranges from 0 to 30 points; higher scores represent a more severe condition^(4)^. Similar to other studies, a score equal to or higher than 13 points was the cutoff point used to indicate the risk of depression^(21-22)^.

Three other forms specifically designed for this study were used. The first collected the participants’ identification information; the second addressed sociodemographic data and information concerning pregnancy planning and acceptance, availability of social support, and the participants’ lifestyle; and the third form addressed data concerning the pregnancy, labor, and childbirth, which were available in the medical records available in the public maternal hospitals located in Botucatu.

### Study variables

The exposure variable was a score indicating depressive symptoms.

**Figure 1:**
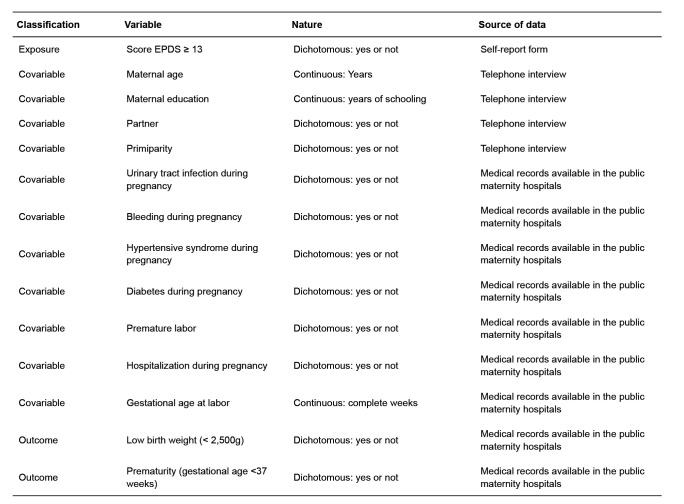
Study’s variables, nature and source of data. Botucatu, SP, Brazil, 2018-2019

Two outcomes were considered: low birth weight and prematurity, and the covariates included maternal sociodemographic factors and complications in the current pregnancy. A synthesis of the variables is presented in Figure 1.

### Data collection

During the recruiting period, all the pregnant women attending prenatal care appointments in the city’s Health Units were invited to participate. The nursing technicians, nurses, and/or physicians working in the Primary Health Care units recruited the participants. The study’s coordinators trained these workers, who were also informed about the study’s objectives and received step-by-step instructions to operationalize data collection. A manual was developed and delivered to each health unit, and the Public Health Research Unit (UPESC) at the Botucatu Medical School remained available to clarify potential doubts.

Data were collected in three stages. Stage 1, after receiving clarification about the study, those who consented to participate received a form containing the EPDS and addressing some identification data. The participants were then conducted to a private room to fill in the forms and later returned the complete forms within a sealed envelope.

Stage 2 was conducted up to 15 days after the pregnant women were included in the cohort. An interview was conducted via telephone to obtain sociodemographic data, and stage 3 consisted of collecting data concerning complications during pregnancy, birth weight, and gestational age at birth from the mothers’ and infants’ medical records available in public maternity hospitals. These last two stages were conducted by undergraduate students and residents from the nursing and nutrition programs, trained and supervised by the Public Health Research Unit team. Periodical meetings were held with the interviewers and the supervising team to support data collection.

Strategies were adopted to avoid a follow-up loss such as asking the participants’ different telephone numbers and those of their partners or relatives; the telephone interview was scheduled on the day the EPDS was applied according to the participants’ convenient day and time, and contact was attempted at least six times before considering it a loss.

### Data analysis

Association between depressive symptoms during pregnancy and the outcomes (low birth weight and prematurity) and the covariates individually associated with the outcomes presenting a p<0.20 were verified. Once identified, these covariates were included in the Cox regression model for adjustments. A critical p<0.05 was established in the multiple analysis to accept the association between the positive score indicating depressive symptoms and low birth weight and prematurity. The Hazard Ratio (HR) was calculated in the crude and adjusted analyzes with a 95% confidence interval, and goodness of fit was assessed through distribution of residuals. Analyses were performed using Statistical Package for the Social Science (SPSS), version 21.

### Ethical aspects

The study was approved by the Research Ethics Board at the Medical School of Botucatu, under protocol No. 2,641,633. The participants signed free and informed consent forms, and whenever depressive symptoms were identified (i.e., equal to or higher than 13), the participants were referred to medical assessment in the health units where they received prenatal care.

## Results

Forty-nine (25.4%) participants presented scores indicating depressive symptoms. Regarding the newborns, 17 were pre-term (8.8%), and 12 presented low birth weight (6.2%); median birth weight was 3,220 g.

Variables concerning maternal sociodemographic characteristics concerning the current pregnancy are presented in Table 1. The pregnant participants were aged 24.9 years old (median) and had 11 years of schooling (median); 82.4% lived with their partners; the frequency of complications during pregnancy ranged from 33.7% with hospitalization, and 5.2% presented bleeding and premature labor; the median gestational age at the time of labor was 39 weeks.

**Table 1:** Maternal sociodemographic characteristics concerning the current pregnancy. Botucatu, SP, Brazil, 2018-2019

Maternal variables	N	%
Live with partner	159	82.4
Primiparity	86	44.6
Urinary tract infection during pregnancy	57	29.5
Bleeding during pregnancy	10	5.2
Hypertensive syndrome during pregnancy	27	14.0
Gestational diabetes	14	7.3
Premature labor	10	5.2
Hospitalization during pregnancy	65	33.7
	Median	Minimum-Maximum
Maternal age (years)	24.9	15.6-45.3
Schooling (years)	11.0	1.0-20.0
Gestational age when completed the EPDS* (weeks)	25.0	4-40
Gestational age at labor (weeks)	39.0	29.0-42.0

* Edinburgh Postnatal Depression Scale

Depression scores did not appear associated with low birth weight (p= 0.530) in the crude analysis (Table 2). The covariates: age, bleeding during pregnancy, premature labor, primiparity, hospitalization during pregnancy, and gestational age at labor were associated with low birth weight (p<0.20) and were included in the multiple analysis together with the variable of interest, depression score. The adjusted model showed no association between depression and low birth weight (p=0.563). Premature labor (p= 0.049) and gestational age at labor (p= 0.017) appeared independently associated with low birth weight. Premature labor increased almost five times (HR=4.81, CI95%=1.0123.0) the risk of low birth weight; each week added to gestational age decreased the risk of low birth weight by 24% (HR=0.76, CI95%= 0.61-0.95).

**Table 2:** Crude and adjusted Cox regression model between a positive score for depression during pregnancy, covariates, and low birth weight. Botucatu, SP, Brazil, 2018-2019

	Raw Analysis			Adjusted Analysis		
Maternal variables	HR*	CI(95%)^†^	p^‡^	HR*	CI(95%)^†^	p^‡^
EPDS score ≥13	1.47	0.44-4.88	0.530	1.49	0.38-5.82	0.563
Age (years)	1.07	0.99-1.16	0.072	1.03	0.93-1.13	0.618
Schooling (years)	0.99	0.73-1.34	0.947			
Live with partner	1.07	0.23-4.88	0.931			
Primiparity	0.25	0.05-1.14	0.073	0.29	0.05-1.61	0.156
Urinary tract infection	1.19	0.36-3.96	0.773			
Bleeding during pregnancy	3.66	0.80-16.70	0.094	5.35	0.73-39.3	0.099
Hypertensive syndrome	1.23	0.27-5.61	0.790			
Diabetes during pregnancy	0.04	0-68.22	0.526			
Premature labor	9.15	2.76-30.39	<0.001^§^	4.81	1.01-23.0	0.049^§^
Hospitalization during pregnancy	2.76	0.88-8.69	0.083	1.15	0.27-4.95	0.856
Gestational age at labor	0.71	0.60-0.84	<0.001^§^	0.76	0.61-0.95	0.017^§^

* Hazard Ratio;

† 95% Confidence Interval;

‡ Level of significance;

§ Result statistically significant.

Table 3 presents the analysis concerning prematurity. The crude analysis (p=0.47) did not show depression scores associated with prematurity. Schooling and premature labor (p≤0.20) were selected as potential confounding factors and included in the adjusted model together with the variable of interest, depression score. The multiple model confirmed the lack of association between prematurity and positive score for depressive symptoms (p=0.826), and showed that premature labor (p<0.001) increased by almost eight times (HR=7.70, CI95%=2.50-23.7) the risk of prematurity, regardless of the level of education or depressive symptoms.

**Table 3:** Crude and adjusted Cox regression model between a positive score for depression during pregnancy, covariates, and prematurity. Botucatu, SP, Brazil, 2018-2019

	Raw Analysis			Adjusted Analysis		
Maternal variables	HR*	IC(95%)^†^	p^‡^	HR*	IC(95%)^†^	p^‡^
EPDS score ≥ 13	0.63	0.18-2.19	0.467	0.87	0.24-3.09	0.826
Age	1.00	0.93-1.08	0.949			
Schooling (years)	1.32	1.11-1.59	0.002^§^	1.11	0.92-1.34	0.254
Live with partner	1.60	0.37-7.01	0.530			
Primiparity	1.40	0.54-3.63	0.489			
Urinary tract infection	1.67	0.64-4.39	0.298			
Bleeding during pregnancy	1.14	0.15-8.62	0.896			
Hypertensive syndrome	0.82	0.19-3.58	0.792			
Gestational diabetes	0.80	0.11-6.03	0.828			
Premature labor	9.98	3.69-26.99	<0.001^§^	7.70	2.50-23.7	<0.001^§^
Hospitalization during pregnancy	1.38	0.52-3.62	0.515			

* Hazard Ratio;

† 95% Confidence Interval;

‡ Level of significance;

§ Result statistically significant.

## Discussion

Contrary to the expected, no association was found in this study between depressive symptoms during pregnancy and low birth weight or prematurity. On the other hand, a large number of pregnant women presented a positive score for depression. This result was similar to that reported by a Brazilian study conducted in another city, which adopted the EPDS and considered a cutoff point of 13 points^(4)^: 27.2%, 21.7%, and 25.4% of the pregnant women with 20, 28, and 36 weeks of pregnancy, respectively presented depression symptoms, corroborating the results reported here.

A convenience sample, composed of pregnant women assisted by the public Primary Health Care service, was used. Thus, it does not represent the city’s entire population, as this limitation prevents the generalization of results. For this reason, future studies addressing larger probabilistic samples are needed. On the other hand, by including only pregnant women cared for by the public health network, the potential effect of high rates of elective C-sections was minimized. C-sections are more common in private services and may influence the risk of low birth weight and prematurity. Therefore, despite the low population representatives, excluding a potential confounding factor (elective C-sections) possibly benefited internal validity. Another factor supporting the results’ validity is that the gestational trimester in which mothers were at the time the EPDS was applied was not associated with the final score or the two outcomes addressed here. Therefore, the fact that the pregnant women were not all assessed in the same stage of pregnancy did not affect the results.

The depression rates reported by international studies among pregnant women vary among countries and within the same country. Using the same scale adopted here and the same cutoff point, studies conducted in China report 28.5%^(23)^, 33.8% in Tanzania^(24)^, and 7.0% in Australia^(25)^. Different rates were found in different places within Ethiopia 11.8%^(26)^, 21.5%^(1)^, and 24.9%^(27)^ so that only the highest rate found in Ethiopian studies was similar to the one reported here. Depressive symptoms identified with the EPDS among pregnant women in Nigeria were similar to those found in this study, though the cutoff point used was 12, limiting comparisons between results^(3)^.

Note that the main result found in this study was no association between depressive symptoms during pregnancy and low birth weight or prematurity. Therefore, the hypothesis that depressive symptoms during pregnancy would increase the risk of these two adverse outcomes was not confirmed. This result is in line with some previous studies^(6-8)^, except for one^(10)^, so that the question remains, especially in Brazil.

Although very plausible, the association between depression during pregnancy and low birth weight and prematurity was not evident in the crude analysis, and other aspects should be considered to explain it. The influence of depression may vary according to the women’s life context. In a situation of extreme poverty, pregnant women with depression tend to experience anorexia, high levels of stress and are less willing to make efforts to obtain proper food and care for their health. This situation may result in low gestational weight gain and a deficiency of nutrients essential for baby growth, consequently leading to low birth weight and prematurity^(28)^. Less poor contexts, in which high-energy food is currently accessible even for lowincome populations, such as in the case of Brazil and other countries in Latin America^(29-30)^, including the city where this study was conducted^(31)^, depression during pregnancy may not negatively influence infants’ birth weight or the risk of prematurity. Therefore, other studies conducted in different Brazilian and international contexts should be conducted to broaden understanding regarding the role of depression on low birth weight and premature labor, confirming or discarding the hypothesis of this context-modifying effect.

Even though no evidence was found that depression affects birth weight or prematurity, the high number of pregnant women with depressive symptoms is relevant due to the potential consequences of this condition on other outcomes during childhood and adolescence. A study conducted with pregnant women with depression reports that these women less frequently attend prenatal care and are more susceptible to consume poor quality food^(32)^, alcohol, and smoking^(3,33-34)^. A literature review addressing the repercussion of maternal depression, including studies conducted in low- and middle-income countries, reports a greater risk of psychological disorders among infants, children, and adolescents; deficient growth and postnatal child development; early weaning; and childhood illnesses, such as diarrhea and other infectious diseases^(35)^.

This study also identified that premature labor increased by approximately five and eight times the risk of low birth weight and prematurity, respectively. On the other hand, each week added to gestational age decreased the risk of low birth weight by 24%. These relations are well known and corroborate the validity of the results reported here.

Premature labor is a significant complication that determines neonatal morbidity and mortality due to the progression to premature childbirth, with consequences in the long term including neurological, cognitive, respiratory, cardiovascular, and psychosocial disorders^(36)^. Studies using varied designs, addressing different populations, and conducted in different countries consistently report an association between premature labor and low birth weight^(37-42)^.

The large portion of women with low-risk pregnancies in this study presenting depressive symptoms indicates that actions should be implemented in health services to identify, manage, and monitor prenatal depression. Considering the vital work of PHC nurses in prenatal care, these professionals are expected to lead actions intended to incorporate routine screening for depression among pregnant women.

## Conclusion

Many women with depressive symptoms were identified in this study. Slightly more than ¼ of the participants obtained scores equal to or higher than 13 in the EPDS. However, no association was found between depressive symptoms during pregnancy and low birth weight or prematurity. On the other hand, premature labor was confirmed as an independent risk factor influencing low birth weight and prematurity, while increased gestational age was confirmed as a protection factor against low birth weight.
